# Poly[[(1,10-phenanthroline-κ^2^ *N*,*N*′)zinc]-μ-2,5-bis­(all­yloxy)terephthalato-κ^2^ *O* ^1^:*O* ^4^]

**DOI:** 10.1107/S1600536811022392

**Published:** 2011-06-18

**Authors:** Ruofei Hu, Yuanying Hu, Qingzeng Zhu

**Affiliations:** a School of Chemistry and Chemical Engineering, Shandong University, Jinan 250100, People’s Republic of China

## Abstract

The title compound, [Zn(C_14_H_12_O_6_)(C_12_H_8_N_2_)]_*n*_, is a coordination polymer forming one-dimensional infinite zigzag chains along [10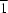] by inter­connection of Zn^II^ atoms by 2,5-bis­(all­yl­oxy)­terephthalate anions *via* the carboxyl­ate groups. The Zn^II^ atom is located on a twofold axis and is in a distorted tetra­hedral coordination formed by the two carboxyl­ate O atoms [Zn—O = 1.9647 (12) Å] and two phenanthroline N atoms [Zn—N = 2.0949 (14) Å].

## Related literature

Some other low-dimensional Zn^II^ complexes based on different organic carb­oxy­lic acids are described by Zhou *et al.* (2009 ▶). For the preparation of 2,5-bis­(all­yloxy)terephthalic acid, see: Kenichiro *et al.* (1998 ▶).
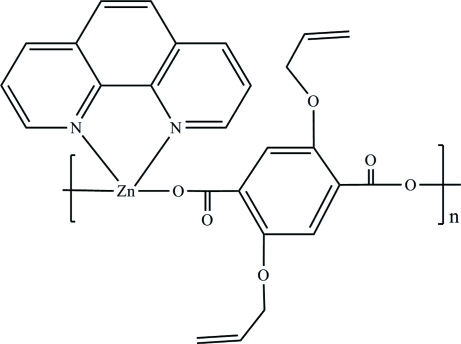


## Experimental

### 

#### Crystal data

[Zn(C_14_H_12_O_6_)(C_12_H_8_N_2_)]
*M* *_r_* = 521.81
Monoclinic, 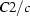

*a* = 21.428 (10) Å
*b* = 9.458 (4) Å
*c* = 12.897 (6) Å
β = 118.462 (5)°
*V* = 2297.9 (18) Å^3^
*Z* = 4
Mo *K*α radiation
μ = 1.12 mm^−1^
*T* = 293 K
0.25 × 0.23 × 0.22 mm

#### Data collection

Bruker *P*4 diffractometer
Absorption correction: multi-scan (*SADABS*; Sheldrick, 1996 ▶) *T*_min_ = 0.768, *T*_max_ = 0.792
8294 measured reflections
2546 independent reflections
2325 reflections with *I* > 2σ(*I*)
*R*_int_ = 0.028
Standard reflections: 0

#### Refinement

*R*[*F* ^2^ > 2σ(*F* ^2^)] = 0.029
*wR*(*F* ^2^) = 0.078
*S* = 1.04
2546 reflections
159 parameters
H-atom parameters constrained
Δρ_max_ = 0.32 e Å^−3^
Δρ_min_ = −0.34 e Å^−3^

### 

Data collection: *APEX2* (Bruker, 2007 ▶); cell refinement: *SAINT-Plus* (Bruker, 2007 ▶); data reduction: *SAINT-Plus*; program(s) used to solve structure: *SHELXS97* (Sheldrick, 2008 ▶); program(s) used to refine structure: *SHELXL97* (Sheldrick, 2008 ▶); molecular graphics: *SHELXTL* (Sheldrick, 2008 ▶); software used to prepare material for publication: *SHELXTL*.

## Supplementary Material

Crystal structure: contains datablock(s) global, I. DOI: 10.1107/S1600536811022392/vn2012sup1.cif

Structure factors: contains datablock(s) I. DOI: 10.1107/S1600536811022392/vn2012Isup2.hkl

Additional supplementary materials:  crystallographic information; 3D view; checkCIF report

## Figures and Tables

**Fig. 1.**
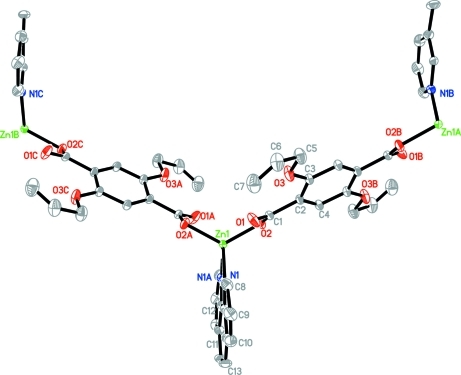
Atom numbering scheme for the title compound with displacement ellipsoids shown at the 30% probability level. [symmetry codes: A, 2 - x, y, 1.5 - z; B, 1.5 - x, 0.5 - y,1 - z; C, 1/2 + x, 0.5 - y, 1/2 + z]

## supplementary crystallographic information

### Experimental

2,5-Bis(allyloxy)terephthalic acid was prepared according to the literature (Kenichiro *et al.*, 1998). Other chemicals were used as purchased. Zn(NO_3_)_2_6(H_2_O) (8.9 mg, 0.03 mmol), 1,10-phenanthroline (1.98 mg, 0.01 mmol) and 2 ,5-bis(allyloxy)terephthalic acid (2.48 mg, 0.01 mmol) were added to the 15 ml N, *N*-dimethylacetamide. After stirring at room temperature for 1 h, the solution was kept at 140 °C for 3 days. Crystallization yielded pink crystals suitable for X-ray diffraction analysis (yield 2.25 mg, 39%).

### Refinement

The methylene H atoms were placed in calculated positions with a C—H bond distance of 0.97 Å  of the carrier atom, and the other  C—H distance were placed at 0.93 Å. *U*_iso_(H) was set at 1.2*U*_eq_ of the carrier atom. Two reflections have been omitted from the refinement as they were most probably partially affected by the beamstop.

### Crystal data

**Table:**

[Zn(C_14_H_12_O_6_)(C_12_H_8_N_2_)]	*F*(000) = 1072
*M**_r_* = 521.81	*D*_x_ = 1.508 Mg m^−^^3^
Monoclinic, *C*2/*c*	Mo *K*α radiation, λ = 0.71073 Å
Hall symbol: -C 2yc	Cell parameters from 80 reflections
*a* = 21.428 (10) Å	θ = 2.4–27.2°
*b* = 9.458 (4) Å	µ = 1.12 mm^−^^1^
*c* = 12.897 (6) Å	*T* = 293 K
β = 118.462 (5)°	Block, pink
*V* = 2297.9 (18) Å^3^	0.25 × 0.23 × 0.22 mm
*Z* = 4	

### Data collection

**Table:**

Bruker P4 diffractometer	2546 independent reflections
Radiation source: fine-focus sealed tube	2325 reflections with *I* > 2σ(*I*)
graphite	*R*_int_ = 0.028
ω scans	θ_max_ = 27.5°, θ_min_ = 3.9°
Absorption correction: multi-scan (*SADABS*; Sheldrick, 1996)	*h* = −27→27
*T*_min_ = 0.768, *T*_max_ = 0.792	*k* = −12→7
8294 measured reflections	*l* = −16→16

### Refinement

**Table:**

Refinement on *F*^2^	Primary atom site location: structure-invariant direct methods
Least-squares matrix: full	Secondary atom site location: difference Fourier map
*R*[*F*^2^ > 2σ(*F*^2^)] = 0.029	Hydrogen site location: inferred from neighbouring sites
*wR*(*F*^2^) = 0.078	H-atom parameters constrained
*S* = 1.04	*w* = 1/[σ^2^(*F*_o_^2^) + (0.0463*P*)^2^ + 0.943*P*] where *P* = (*F*_o_^2^ + 2*F*_c_^2^)/3
2546 reflections	(Δ/σ)_max_ = 0.001
159 parameters	Δρ_max_ = 0.32 e Å^−^^3^
0 restraints	Δρ_min_ = −0.34 e Å^−^^3^

### Special details

**Table:**

Geometry. All e.s.d.'s (except the e.s.d. in the dihedral angle between two l.s. planes) are estimated using the full covariance matrix. The cell e.s.d.'s are taken into account individually in the estimation of e.s.d.'s in distances, angles and torsion angles; correlations between e.s.d.'s in cell parameters are only used when they are defined by crystal symmetry. An approximate (isotropic) treatment of cell e.s.d.'s is used for estimating e.s.d.'s involving l.s. planes.
Refinement. Refinement of *F*^2^ against ALL reflections. The weighted *R*-factor *wR* and goodness of fit *S* are based on *F*^2^, conventional *R*-factors *R* are based on *F*, with *F* set to zero for negative *F*^2^. The threshold expression of *F*^2^ > σ(*F*^2^) is used only for calculating *R*-factors(gt) *etc*. and is not relevant to the choice of reflections for refinement. *R*-factors based on *F*^2^ are statistically about twice as large as those based on *F*, and *R*- factors based on ALL data will be even larger.

### Fractional atomic coordinates and isotropic or equivalent isotropic displacement parameters (Å^2^)

**Table:**

	*x*	*y*	*z*	*U*_iso_*/*U*_eq_	
Zn1	1.0000	0.52903 (2)	0.7500	0.02792 (10)	
O1	0.89786 (8)	0.40767 (17)	0.77262 (12)	0.0557 (4)	
O2	0.91910 (6)	0.43004 (14)	0.62292 (11)	0.0391 (3)	
O3	0.80413 (7)	0.19659 (16)	0.73891 (11)	0.0514 (4)	
N1	0.97095 (7)	0.69874 (13)	0.82237 (11)	0.0301 (3)	
C1	0.88075 (8)	0.38949 (16)	0.66788 (14)	0.0336 (3)	
C2	0.81280 (8)	0.31540 (16)	0.58400 (14)	0.0312 (3)	
C3	0.77669 (8)	0.22152 (18)	0.62095 (14)	0.0337 (3)	
C4	0.78541 (8)	0.34259 (17)	0.46410 (15)	0.0345 (3)	
H4	0.8092	0.4055	0.4398	0.041*	
C5	0.76293 (11)	0.1225 (3)	0.78059 (18)	0.0574 (5)	
H5A	0.7644	0.0218	0.7674	0.069*	
H5B	0.7139	0.1533	0.7381	0.069*	
C6	0.79184 (12)	0.1506 (3)	0.90851 (19)	0.0650 (6)	
H6	0.7738	0.0967	0.9484	0.078*	
C7	0.83955 (14)	0.2426 (3)	0.9693 (2)	0.0753 (7)	
H7A	0.8592	0.2989	0.9331	0.090*	
H7B	0.8545	0.2528	1.0494	0.090*	
C8	0.94114 (9)	0.6966 (2)	0.89252 (16)	0.0409 (4)	
H8	0.9341	0.6100	0.9195	0.049*	
C9	0.92019 (11)	0.8196 (2)	0.92674 (18)	0.0503 (5)	
H9	0.8989	0.8143	0.9748	0.060*	
C10	0.93107 (11)	0.9478 (2)	0.88954 (18)	0.0482 (5)	
H10	0.9163	1.0302	0.9108	0.058*	
C11	0.96472 (10)	0.95499 (17)	0.81881 (15)	0.0390 (4)	
C12	0.98315 (8)	0.82583 (15)	0.78693 (13)	0.0290 (3)	
C13	0.98266 (11)	1.0847 (2)	0.78170 (17)	0.0508 (5)	
H13	0.9700	1.1703	0.8020	0.061*	

### Atomic displacement parameters (Å^2^)

**Table:**

	*U*^11^	*U*^22^	*U*^33^	*U*^12^	*U*^13^	*U*^23^
Zn1	0.02088 (14)	0.02229 (15)	0.03263 (15)	0.000	0.00630 (10)	0.000
O1	0.0577 (8)	0.0624 (9)	0.0399 (7)	−0.0299 (7)	0.0174 (6)	−0.0169 (6)
O2	0.0269 (5)	0.0430 (6)	0.0418 (6)	−0.0118 (5)	0.0118 (5)	−0.0097 (5)
O3	0.0367 (7)	0.0751 (10)	0.0331 (6)	−0.0212 (6)	0.0091 (5)	0.0021 (6)
N1	0.0270 (6)	0.0291 (6)	0.0329 (6)	−0.0017 (5)	0.0132 (5)	−0.0011 (5)
C1	0.0248 (7)	0.0271 (8)	0.0398 (8)	−0.0029 (6)	0.0081 (6)	−0.0056 (6)
C2	0.0214 (7)	0.0311 (8)	0.0365 (8)	−0.0030 (5)	0.0101 (6)	−0.0066 (6)
C3	0.0246 (7)	0.0370 (8)	0.0344 (8)	−0.0036 (6)	0.0099 (6)	−0.0026 (6)
C4	0.0252 (7)	0.0353 (8)	0.0393 (8)	−0.0064 (6)	0.0124 (6)	−0.0027 (6)
C5	0.0450 (11)	0.0793 (15)	0.0469 (11)	−0.0162 (10)	0.0210 (9)	0.0023 (10)
C6	0.0535 (12)	0.0975 (19)	0.0473 (11)	−0.0056 (12)	0.0266 (10)	0.0018 (12)
C7	0.0639 (15)	0.103 (2)	0.0546 (13)	−0.0002 (14)	0.0251 (12)	−0.0175 (14)
C8	0.0395 (9)	0.0452 (10)	0.0427 (9)	−0.0084 (7)	0.0235 (8)	−0.0055 (7)
C9	0.0469 (10)	0.0625 (13)	0.0518 (11)	−0.0080 (9)	0.0320 (9)	−0.0180 (9)
C10	0.0451 (10)	0.0499 (11)	0.0482 (10)	0.0069 (8)	0.0211 (9)	−0.0151 (8)
C11	0.0418 (9)	0.0318 (9)	0.0348 (8)	0.0056 (7)	0.0113 (7)	−0.0054 (6)
C12	0.0271 (7)	0.0263 (7)	0.0275 (7)	0.0009 (5)	0.0082 (6)	−0.0016 (5)
C13	0.0718 (14)	0.0241 (8)	0.0441 (10)	0.0068 (8)	0.0175 (9)	−0.0022 (7)

### Geometric parameters (Å, °)

**Table:**

Zn1—O2	1.9647 (12)	C5—H5A	0.9700
Zn1—O2^i^	1.9647 (12)	C5—H5B	0.9700
Zn1—N1	2.0949 (14)	C6—C7	1.285 (4)
Zn1—N1^i^	2.0950 (14)	C6—H6	0.9300
O1—C1	1.232 (2)	C7—H7A	0.9300
O2—C1	1.270 (2)	C7—H7B	0.9300
O3—C3	1.365 (2)	C8—C9	1.392 (3)
O3—C5	1.418 (2)	C8—H8	0.9300
N1—C8	1.333 (2)	C9—C10	1.365 (3)
N1—C12	1.355 (2)	C9—H9	0.9300
C1—C2	1.510 (2)	C10—C11	1.408 (3)
C2—C4	1.392 (2)	C10—H10	0.9300
C2—C3	1.402 (2)	C11—C12	1.404 (2)
C3—C4^ii^	1.397 (2)	C11—C13	1.434 (3)
C4—C3^ii^	1.397 (2)	C12—C12^i^	1.444 (3)
C4—H4	0.9300	C13—C13^i^	1.342 (4)
C5—C6	1.484 (3)	C13—H13	0.9300
			
O2—Zn1—O2^i^	123.08 (8)	C6—C5—H5B	109.9
O2—Zn1—N1	113.91 (6)	H5A—C5—H5B	108.3
O2^i^—Zn1—N1	108.96 (6)	C7—C6—C5	126.0 (2)
O2—Zn1—N1^i^	108.96 (6)	C7—C6—H6	117.0
O2^i^—Zn1—N1^i^	113.91 (6)	C5—C6—H6	117.0
N1—Zn1—N1^i^	79.98 (8)	C6—C7—H7A	120.0
C1—O2—Zn1	105.59 (11)	C6—C7—H7B	120.0
C3—O3—C5	119.50 (14)	H7A—C7—H7B	120.0
C8—N1—C12	118.32 (14)	N1—C8—C9	122.23 (17)
C8—N1—Zn1	129.09 (11)	N1—C8—H8	118.9
C12—N1—Zn1	112.55 (10)	C9—C8—H8	118.9
O1—C1—O2	122.65 (14)	C10—C9—C8	119.77 (16)
O1—C1—C2	122.14 (15)	C10—C9—H9	120.1
O2—C1—C2	115.21 (14)	C8—C9—H9	120.1
C4—C2—C3	119.07 (14)	C9—C10—C11	119.75 (16)
C4—C2—C1	117.55 (14)	C9—C10—H10	120.1
C3—C2—C1	123.39 (15)	C11—C10—H10	120.1
O3—C3—C4^ii^	122.92 (15)	C12—C11—C10	116.75 (16)
O3—C3—C2	118.29 (14)	C12—C11—C13	119.27 (17)
C4^ii^—C3—C2	118.79 (15)	C10—C11—C13	123.96 (16)
C2—C4—C3^ii^	122.14 (15)	N1—C12—C11	123.14 (14)
C2—C4—H4	118.9	N1—C12—C12^i^	117.39 (8)
C3^ii^—C4—H4	118.9	C11—C12—C12^i^	119.47 (10)
O3—C5—C6	109.10 (18)	C13^i^—C13—C11	121.18 (11)
O3—C5—H5A	109.9	C13^i^—C13—H13	119.4
C6—C5—H5A	109.9	C11—C13—H13	119.4
O3—C5—H5B	109.9		
			
O2^i^—Zn1—O2—C1	78.15 (10)	C3—C2—C4—C3^ii^	−0.5 (3)
N1—Zn1—O2—C1	−57.33 (12)	C1—C2—C4—C3^ii^	179.32 (15)
N1^i^—Zn1—O2—C1	−144.52 (10)	C3—O3—C5—C6	−160.82 (19)
O2—Zn1—N1—C8	72.39 (16)	O3—C5—C6—C7	9.4 (4)
O2^i^—Zn1—N1—C8	−69.20 (15)	C12—N1—C8—C9	2.2 (3)
N1^i^—Zn1—N1—C8	178.81 (18)	Zn1—N1—C8—C9	−175.24 (14)
O2—Zn1—N1—C12	−105.20 (11)	N1—C8—C9—C10	−1.0 (3)
O2^i^—Zn1—N1—C12	113.21 (11)	C8—C9—C10—C11	−1.3 (3)
N1^i^—Zn1—N1—C12	1.21 (7)	C9—C10—C11—C12	2.2 (3)
Zn1—O2—C1—O1	−3.4 (2)	C9—C10—C11—C13	−175.80 (19)
Zn1—O2—C1—C2	177.38 (10)	C8—N1—C12—C11	−1.2 (2)
O1—C1—C2—C4	157.66 (17)	Zn1—N1—C12—C11	176.68 (12)
O2—C1—C2—C4	−23.1 (2)	C8—N1—C12—C12^i^	178.70 (17)
O1—C1—C2—C3	−22.5 (2)	Zn1—N1—C12—C12^i^	−3.4 (2)
O2—C1—C2—C3	156.76 (15)	C10—C11—C12—N1	−1.0 (2)
C5—O3—C3—C4^ii^	−11.2 (3)	C13—C11—C12—N1	177.11 (15)
C5—O3—C3—C2	169.03 (18)	C10—C11—C12—C12^i^	179.11 (18)
C4—C2—C3—O3	−179.70 (15)	C13—C11—C12—C12^i^	−2.8 (3)
C1—C2—C3—O3	0.5 (2)	C12—C11—C13—C13^i^	−0.7 (3)
C4—C2—C3—C4^ii^	0.5 (3)	C10—C11—C13—C13^i^	177.2 (2)
C1—C2—C3—C4^ii^	−179.32 (14)		

Symmetry codes: (i) −*x*+2, *y*, −*z*+3/2; (ii) −*x*+3/2, −*y*+1/2, −*z*+1.
